# On Simple Ulceration of the Os Uteri

**Published:** 1845-07

**Authors:** 


*On Simple Ulceration of the Os Uteri.*—The occurrence of simple ulcers on the os uteri was denied by Boyer, owing, no doubt, to the little use made of the speculum in his time. Nothing, however, is more frequent than the appearance of these ulcers; and it may be said that every woman labouring under leucorrhœa, purulent or lactescent, is affected by this disease, if not with cancer. Five or six varieties of this disease are at present under treatment in the wards of M. Jobert, at the Hôpital St. Louis; and these we have carefully studied by means of the speculum. It is so rare in ordinary practice, to have an equal number of patients under the eye at one time, and so inconvenient, moreover, to examine them in a suitable manner, that the present opportunity of doing so is interesting. The disease, as far as regards the ulceration, presents itself under various forms; but they all proceed from the same cause, hypertrophy of the neck. This hypertrophy, without doubt, precedes the erosion, and is sometimes accompanied with induration, sometimes with softening. The hypertrophic softening is sometimes considerable; we have seen the neck undulate, and even yield under the simple pressure of a pencil of agaric, like a stewed apple. In this condition the neck, from the absence of nerves in its tissue, presents no morbid sensibility. The ulceration appears, no doubt, consecutively to this state, and is the natural process of chronic inflammation. The ulcers may have their seat on one or other lip, generally the superior, sometimes on both, and very frequently on one or the other side; in some cases they cover the whole circle of the os, and in others they have their seat deep in the neck of the uterus, where they are concealed by the swelling of the anterior lip; but they may be discovered there by a means which we shall presently indicate: so much for the seat of the ulcers. As to their form, they are sometimes superficial,—simple aphthæ,—of the size of a lentil, having their seat in the edge of the neck, and more or less numerous; this is the most simple case; these aphthæ, however, sometimes extend, become confounded together, and constitute a superficial erosion of more or less extent of a mapped form, and more or less irregular; the lesion then becomes much more serious. It is not necessary, however, that it should pass through the aphthous stage to arrive at this state; for it may originate primarily and to a great extent, from the inflammatory process alone. This species of ulceration presents a great resemblance to those large erosions of the superior part of the cornea, in form of a cross, described by Demours, and styled by Velpeau "ulcères à coup d'ongle;" it is, however, proportionally much larger. It may be compared more exactly to the surface of a suppurating blister; it is sometimes covered with granulations, bleeds easily, and is often even infiltrated with blood; thus its aspect is always red; it is not painful to the touch, either with the finger or a pencil. It is probable that these women, in whom there is hemorrhage after sexual intercourse, have some slight ulcerative lesion of this kind. In a third variety, the erosion is no longer a mere superficial excoriation, it is hollow, infundibuliform, semi-spherical, more or less deep, sometimes very deep. Its base is more or less foul; its surface is always of a bright red, and infiltrated with blood. The erosion then very much resembles certain hollow ulcers of the legs in varicose subjects, who have just been walking. This kind of ulcer often causes a notch on one side of the os uteri, near its opening or on its free edge, but more generally on its superior lip, or towards the left lateral commissure. In some cases it affects the whole circle of the internal surface of the os, and hollows out a progressive cavity from above downwards. These hollow erosions must always be regarded with suspicion, more especially if they make any progress in depth, for their nature is frequently not simple; and if they have been so, they are liable to assume a bad character. It may be said, generally, that the ulcer is simple when its surface is granular. In regard to the third variety, it resembles the two preceding as to form, only it has its seat in the neck. In conclusion, we have thus observed three varieties of simple ulcers on the neck of the uterus; the aphthous, ulcerative abrasions, and hollow ulcers; they are all hemorrhagic, especially the latter, and are more or less granular. Hollow ulcers, not granular, are always to be regarded with suspicion. We have not included syphilitic sores of the neck, primary or secondary; these lesions do not in general exist alone, and they have, moreover, specific characters, which at present we need not mention.

Those affected with ulceration of the neck of the uterus are generally young, having seldom passed their thirtieth year; have usually had a family, or miscarriages, and have been for some time subject to abundant leucorrhœa, and hemorrhages, or at least to fluxes of blood from the uterus other than the catamenial. Their constitution is often lymphatic, but this has not appeared to us predominant. They are frequently dark women, with large black eyes, robust, ardent, in whom the crineous system is much developed, indicating a great degree of of vigour in the vitality of the dermic covering. These conditions may perhaps be regarded as predisposing causes of the hypertrophy, and the consequent ulcerations, owing to the congestive state of the skin, the mucous linings, and the neighbouring organs associated with it; these, however, are mere conjectures.

The patients affected with this disease present two kinds of symptoms. On the one hand, an abundant leucorrhœa, with a lactescent discharge; on the other, symptomatic phenomena peculiar to most of the chronic affections of the uterus; viz. lassitude of the extremities, pain and dragging in the loins, hips and thighs, want of appetite, and sometimes a painful spasmodic contraction of the sphincter ani. These symptoms are accompanied with general languor, more or less troublesome.

A precise diagnosis can only be obtained by means of an accurate examination with the speculum. The "*toucher*" alone is not sufficient; a state of hypertrophy merely can be ascertained by its means, but even then its degree can never be perfectly and clearly defined, however expert the examiner may be. In order to institute a thorough examination with the speculum, the patient must be placed, not on the edge of the bed, as is usually done, but on a table, with the hips very much raised, and the thighs bent backwards, so that the knees almost touch the abdomen. It is then only by means of a strong ray of natural light that the fundus of the vagina can be distinctly seen. In order to examine the whole periphery of the neck, a double-valved speculum ought to be used, which on opening embraces it entirely. A single cylindrical speculum is not so serviceable for the first examination, as its opening does not include the whole hypertrophied mass. At first there is observed on the neck and fundus of the vagina, a quantity of purulent mucus; on wiping it away by means of an agaric pencil, the disease is then visible. The first thing that strikes the eye is hypertrophy of one or other lip, or of the whole of the os, and then the ulcerations with which it is complicated. When there is hypertrophy, with pus in the passage of the neck, ulceration, which is not visible, may be suspected. The following is the method which M. Jobert employs to discover this:—He withdraws the double speculum, and introduces the cylindrical one in one piece, and manœuvres it in such a way as to engage the os tincæ in the centre of its opening; he then inclines the handle of the instrument obliquely to the right or left, or from above downwards, in such a way as to cause the posterior opening of the speculum to slide in the opposite manner on the neck; he thus places one of the lips of the os on the edge of the opening of the speculum, and then pushes the instrument from above downwards, so as to separate the lips, which, from the softness of the tissues, is easily accomplished; a considerable portion of the neck then becomes visible, and the ulcerations are brought into view. These ulcers are generally very small, (like a lentil) but, so far as they extend, are as readily seen as the others. When they are simple, their tendency is to progress from the interior outwards, rather than in the opposite way.

As to the *treatment*, nothing is more simple or certain. The disease is invariably cured in the course of a few months, by the means employed at St. Louis. Two lesions have to be considered, the one dependent on the other, viz: ulceration and hypertrophy. If there are merely aphthous ulcerations, slight cauterisation with the acid nitrate of mercury, or even with the nitrate of silver, speedily produces cicatrization; the remaining hypertrophy, if it is not considerable, may be cured by the ordinary means. If it be to a great degree, the actual cautery is used for both lesions from the commencement. The latter treatment is also employed when the hypertrophy of the neck, though not considerable, is obstinate, and the leucorrhœa continues. The actual cautery is used for the other species of ulcers either by reverberation, or, which is more general, by its direct application to the ulcer, so as to produce an eschar more or less deep; it may be repeated in the course of eight or fifteen days. The cure is generally accomplished in the course of two, three, or four months; there is melioration in regard to the pain and leucorrhœa during the first week. It seems probable, that concentrated heat causes such a modification in the diseased tissues, as to dispose to a healing process. We earnestly entreat attention to the above facts: the disease is both frequent and disastrous among all classes, and more especially in large towns.—*Lond. and Ed. Monthly Journ., from Annales de Therapeutique.*

